# The Causal Effect of Parent–Child Interactions on Child Language Development at 3 and 4 Years

**DOI:** 10.1111/1460-6984.70045

**Published:** 2025-05-05

**Authors:** Mary E. Brushe, Murthy N. Mittinty, Tess Gregory, Dandara Haag, John W. Lynch, Sheena Reilly, Edward Melhuish, Sally A. Brinkman

**Affiliations:** ^1^ The Kids Research Institute Australia University of Western Australia Adelaide Australia; ^2^ School of Public Health University of Adelaide Adelaide Australia; ^3^ College of Medicine and Public Health Flinders University Bedford Park, SA Australia; ^4^ Population Health Sciences, Bristol Medical School University of Bristol Bristol UK; ^5^ Menzies Health Institute Queensland Griffith University Gold Coast Australia; ^6^ Department of Education University of Oxford Oxford UK; ^7^ Education Futures University of South Australia City West Australia

**Keywords:** children, interaction, language development, longitudinal, parents

## Abstract

**Background:**

Language development is critical for children's life chances. Promoting parent–child interactions is suggested as one mechanism to support language development in the early years. However, limited evidence exists for a causal effect of parent–child interactions on children's language development.

**Methods:**

Data from the Language in Little Ones study, an Australian prospective birth cohort study (*n* = 296), was used to determine the sustained effect of parent–child interactions over time on children's language development at 36 and 48 months, measured using the Clinical Evaluation of Language Fundamentals Preschool‐2 (CELF‐P2) language assessment. Marginal structural models and inverse probability of treatment weights were used to allow observational data to emulate a randomised controlled trial by accounting for time‐varying exposures and confounding. These results were then used to estimate the effect of several hypothetical scenarios where the exposure was fixed for the whole population at different levels (5th, 25th, 50th, 75th and 95th percentile) across the observed distribution of parent–child interactions.

**Results:**

Findings supported a causal effect of parent–child interactions from 6 to 36 (or 48) months on children's language development at 36 and 48 months, in a population of children without language impairment. The counterfactual language score at 48 months increased from 97.21 (95% CI 96.86, 97.56) for the scenario fixed at the 5th percentile to 102.15 (95% CI 101.80, 102.50) at the 50th percentile and 111.41 (95% CI 111.06, 111.76) at the 95th percentile.

**Conclusions:**

Although the effects of parent–child interactions on later language were small they do offer one mechanism to support early language development. These findings are discussed within the context of existing interventions to highlight the value of investment into sustained, universal prevention efforts for supporting early language.

**WHAT THIS PAPER ADDS:**

*What is already known on the subject*

- Promoting parent–child interactions within the home environment has been previously suggested as one mechanism to support children's early language development. Nonetheless, there is a lack of causal evidence and long‐term follow‐up to support this claim.

*What this paper adds to the existing knowledge*

- The effect of parent–child interactions throughout the early years on children's language development is explored using causal inference methodology within an Australian prospective birth cohort study. Findings show a small causal effect of increasing parent–child interactions on children's language development at 36 and 48 months, after controlling for time‐varying exposures and confounders.

*What are the potential or actual clinical implications of this work?*

- This highlights the value of sustained, universal early intervention, which encourages back‐and‐forth parent–child interactions, as early as possible. Practitioners who work with parents and carers in the first year of a child's life should promote the importance of talking and interacting with their child to improve later language outcomes.

## 1 Introduction

A child's language acquisition is a critical developmental milestone affecting academic, mental health and employment outcomes into adulthood (Law et al. 2009). Unfortunately, many children start school without the requisite language skills to succeed (Department for Education SaE 2022; Reilly et al. 2010; Dockrell and Hurry 2018), resulting in a greater policy focus on universal prevention services to support children's early language development (Law et al. 2017; Law et al. 2013; Law and Levickis 2018). A recent systematic review of child language interventions in the early years has highlighted the need to move away from clinic‐based interventions that focus on children with existing language difficulties to a public health approach that allows for preventative efforts (Levickis et al. 2022). Such services have often focused on improving the home language environment of young children (Walker and Carta 2020). One mechanism that has been shown to improve child language outcomes is increasing parent–child interactions, whereby parents engage in back‐and‐forth conversations with the child (Levickis et al. 2022).

Despite acknowledgement of the need for universal interventions to promote child language development, limited evidence exists for children under 3 years old (Levickis et al. 2022; McKean and Reilly 2023). The evidence for universal interventions focused on promoting the quantity of parent–child interactions for typical developing children has shown promising short‐term increases in the number of conversational turns between adult and child (Ferjan Ramírez et al. 2019; Beecher and Van Pay 2020). However, there is limited evidence to support sustained increases over time. One US study that targeted mother–toddler dyads from low socioeconomic backgrounds (*n* = 149) trialled a home visiting intervention from 14 to 20 months old and demonstrated sustained increases in the quantity and quality of maternal language input at 26, 32 and 38 months (Leung et al. 2022). However, child language was not measured at follow‐up, making it impossible to determine whether the intervention improved children's longer‐term language outcomes (Bailey et al. 2017). The limited evidence supporting universal interventions, particularly the lack of long‐term follow‐up to demonstrate the return on investments, hinders public health efforts to promote investment in early language interventions.

Randomised controlled trials (RCTs) are well established as the gold‐standard method to determine the causal effects of an intervention (Jones and Podolsky 2015). Nonetheless, the feasibility, cost and resource intensity of these trials can make long‐term follow‐up difficult (Hernán and Robins 2016). Fortunately, in scenarios where RCTs are unethical, unfeasible or too costly, epidemiological methods allow for observational data, when designed with a correct data structure (e.g., data on confounders are collected), to be used to make causal inferences under specific theoretical assumptions (see Supporting Information Appendix for assumptions) (Hernán et al. 2019). The potential outcomes framework (Rubin 2005) offers a way to estimate causal effects by answering ‘what if’ questions about the potential outcomes if an individual had been exposed or unexposed to a particular event.

This study uses data from a prospective cohort study and employs epidemiological methods to answer the policy‐relevant question of whether increasing parent–child interactions over time improves children's language development. Using marginal structural models, we estimate the joint average treatment effect (ATE, see Section 2 for definition) of time‐varying exposures (parent–child interactions) on child language development at 36 and 48 months, after accounting for baseline and time‐varying confounders. These estimates create a pseudo population that is used to model five hypothetical scenarios whereby exposure to the number of parent–child interactions is fixed for the whole population at specific levels of the observed data over time to determine the causal effects of scenarios on a child's language development.

## 2 Methods

### 2.1 Study Design

The Language in Little Ones (LiLO) study is a prospective study that collected data on the child's home language environment every 6 months, from 6 to 48 months old. At each wave of data collection, the parent answered questions about child and family characteristics and completed a Language ENvironment Analysis (LENA) recording day. At 36 and 48 months old, a language assessment was conducted with the child. Ethics approvals were granted by the University of Western Australia Human Research Ethics Committee (2022/ET000028).

### 2.2 Participants

Families were approached to participate at recruitment locations across three Australian states: South Australia, Western Australia and Queensland. Recruitment sites included public hospitals, child and family health services, council‐run immunisation clinics, playgroups and libraries. Eligibility criteria specified that children were born in 2017, were full‐term gestation (>37 weeks), a singleton birth, English was the main language spoken at home and did not have a diagnosed cause of language impairment (e.g., cerebral palsy). At each wave, parents were asked if the child had received a new diagnosis that may affect their language development; if this occurred, they became ineligible. An explicit goal of the LiLO study was to examine socioeconomic differences in early language exposure, and so families were recruited from two levels of maternal education: a lower‐educated group (mothers without any post‐secondary school qualification) and a higher‐educated group (mothers with a university degree). There was difficulty in recruiting families for the lower‐educated group (Galea and Tracy 2007), which led to continued recruitment after Wave 1 to increase participation among this group.

A total of 302 families participated in at least one wave of data collection. Over half (*n* = 166, 56.1%) had missing data on at least one variable throughout the study (see Figure S2). Missingness was due to participants joining the study after Wave 1, attrition over time, participants skipping certain waves, and children or parents opting out of specific questionnaires or assessments (Table S1 summarises missing data).

### 2.3 Measures

The exposure, parent–child language interactions, was a continuous variable of the number of conversational turns undertaken by the adult and child per day as measured by LENA (Gilkerson and Richards; Xu et al. 2009), for each wave of data collection. LENA comprises a digital language processor worn by the child in the pocket of a t‐shirt, which records all the audio within the child's environment over a 16‐h recording day. LENA software then analyses the speech's acoustic properties to estimate the quantity of conversational turns. High levels of agreement (82% for adult words; 76% for child vocalisations) between the LENA software and human transcribers exist within similar English‐speaking populations (Xu et al. 2009). The conversational turn count is based on the number of alternations between adult and child occurring within 5s of each other, initiated by either adult or child.

The outcome, child language development, was assessed using the Clinical Evaluation of Language Fundamentals Preschool‐2 (CELF‐P2) (Wiig et al. 2006) core language standard score at Waves 6 (36 months) and 8 (48 months). The core language score comprises three subtests – sentence structure, word structure and expressive vocabulary – and has been standardised for an Australian and New Zealand population. The continuous age‐corrected standard score was used, which has a potential range of 45–145 (SD = 15) and a score of 100 translating to average development for children of the same age.

Child and family characteristics determined a priori to be associated with the exposure and outcome (i.e., confounders) are depicted in a directed acyclic graph (DAG) (Tennant et al. 2020) (see Figure S1). Baseline confounders included: child gender, highest level of maternal education, child first born status and primary caregivers’ language ability (Reilly et al. 2010; Rowe et al. 2005; Rinaldi et al. 2023). Time‐varying confounders included: the average number of home activities completed with the child, the number of children's books in the home, primary caregivers’ mental health, the financial stability of the family and the amount of time the child was exposed to a screen (Reilly et al. 2010; Rowe et al. 2005; Christakis et al. 2009; Zimmerman et al. 2007; Lovejoy et al. 2000; Glasheen et al. 2010; Hayes et al. 2018). All variables were parent‐reported to the researcher during the biannual home visit, except the measure of parent language ability, which was assessed using the NIH Toolbox version 2.0 Picture Vocabulary Test (Northwestern University, National Institutes of Health 2015), and the child's screen exposure, which was captured through manual coding of the LENA technology (Brushe et al. 2023; Brushe et al. 2024). See Supporting Information for a detailed description of measures used for each confounding variable.

### 2.4 Data Collection

The eight waves of the study were conducted between 2017 and 2022. Researchers attended participants’ homes, where they demonstrated how to use the LENA equipment and completed questionnaires with the primary caregiver. When children were 36 and 48 months, the CELF‐P2 language assessment was completed. The LENA equipment was then left with the family to find an appropriate ‘recording day’. Families were advised that this should not be a day the child was sick, at childcare or attending a large public event. Approximately two weeks later, the researcher returned to collect the equipment and provide reimbursement. This procedure remained consistent across all waves, except during the COVID‐19 pandemic. Government‐imposed lockdowns, beginning in March 2020, meant parents were provided the option of moving ‘home visits’ online through phone or video calls, and the LENA equipment was posted to the family with a paid postbag included to return the equipment. Once the equipment was returned, reimbursement was posted to the family. This occurred for some participants during Waves 5, 6 or 7 depending on the timing of their milestone and residential location, as restrictions fluctuated throughout 2020 and 2021.

### 2.5 Statistical Analysis

The goal of this study was to estimate the joint ATE of the exposure $\bar{A}\;$(parent–child interactions at each time point) on the outcome Y (child's language development at the final time point). The joint ATE is defined by a collection of direct effects: (1) the direct effect of $A_{1}$ on Y, unmediated by $\left\{A_{2},\text{…},A_{T}\right\}$; (2) the direct effect of $A_{2}$ on Y, unmediated by $\left\{A_{3},\text{…},A_{T}\right\}$; and so on for each wave of data collection (Daniel et al. 2013). Estimating the joint ATE supports answering the policy‐relevant question: does increasing parent–child interactions over time improve children's language development at 36 and 48 months?

Missing data were imputed assuming that the data are missing at random (Little and Rubin 2002), with the ‘*mi impute chained*’ command in Stata version 17 (StataCorp 2021). The imputation model included the exposures, outcome, baseline and time‐varying confounders, and auxiliary variables to improve the accuracy of imputation (e.g., child's gestation, mother's age at childbirth). Twenty imputed datasets with 50 iterations were generated, with one imputed dataset being selected at random for statistical analysis. This approach is regarded as acceptable by methodologists when dealing with time‐varying exposures and confounders, given standard errors are not estimated analytically, but in this case, using the simulated distributions (Daniel et al. 2011).

#### 2.5.1 Scenario Setting of the Joint Average Treatment Effect

A two‐stage estimation process was used to estimate the joint ATE. Firstly, we used marginal structural mean models (MSMs), given our study has repeated exposures over time $\left(A_{t}\right)$, baseline confounders (C), time‐varying confounders $\left(L_{t}\right)$, and $L_{t}$ is also affected by $A_{t-1}$ (i.e., the number of home activities at one wave is likely influenced by the number of parent–child interactions at a previous wave) (see DAG in Figure S1). When an arrow is present from $A_{\left(t-1\right)}\to L_{t}$, standard regression approaches may retrieve biased results, so MSM models were employed, using inverse probability of treatment weights (IPTWs), to reduce this bias (Daniel et al. 2013; Robins et al. 2000). Details are provided in the Supporting Information.

Separate MSM models were fit for the outcome at two ages, 36 and 48 months. Model 1 used data from 6 to 36 months, with language development measured at 36 months. Model 2 uses data from 6 to 48 months, with language development measured at 48 months and language development at 36 months was a time‐varying confounder. The MSM estimates reflect the mean change in the child's language development score that would have been observed at 36 (or 48 months), if every child was exposed to one additional parent–child interaction at the specific time point, holding all prior and future exposures, and baseline confounders constant. These estimates create a pseudo population, which is then used in step two, to answer our question concerning the joint ATE.

In step two, we used the coefficients of the pseudo population from the MSM model and calculated the joint ATE, by standardising the counterfactual language development score under a specific trajectory of the joint exposures (i.e., parent–child interactions at each time‐point), across the baseline covariates (see Supporting Information). Typically, binary exposures are used in potential outcome research, which allows for easy interpretation of hypothetical interventions where a population is exposed or unexposed. However, there is no theoretical consensus about an appropriate binary cut‐point for the number of parent–child interactions at different ages. Therefore, Table 2 summarises the five hypothetical scenarios that were modelled based on shifting the number of parent–child interactions the entire population was exposed to from the 5th percentile to the 25th, 50th, 75th and the 95th percentile of the observed distribution of parent–child interactions in our data. Therefore, the joint ATE can be interpreted as the average language development score if all children received parent–child interactions at the level of the *X*th percentile from 6 to 36 (or 48) months. The joint ATE was estimated for five different exposure trajectories, separately for Models 1 and 2. Analyses were conducted in Stata version 17 (StataCorp 2021).

## 3 Results

The final imputed sample included 296 families (Figure S2), with all estimates using this sample. Table 1 summarises child and family characteristics of the sample (see Table S2 for characteristics of the eligible sample before imputation). The average language development score was 103.8 (SD = 12.5) at 36 months and 103.5 (SD = 12.1) at 48 months. Table 2 presents the distribution of parent–child interactions at each wave, with interactions increasing from an average of 331 (SD = 137) at 6 months to 741 (SD = 388) at 48 months.

**TABLE 1:** Child and family characteristics of the imputed sample (*n* = 296).

	Imputed sample
Child	
Girls, *n* (%)	157 (53.0)
Gestation, week, mean (SD)	39.3 (1.5)
Firstborn, *n* (%)	148 (50.0)
Language development standard score at 36 months, mean (SD)	103.8 (12.5)
Language development standard score at 48 months, mean (SD)	103.5 (12.1)
Mother	
Highest level of completed education, university, *n* (%)	166 (56.1)
Age at childbirth, year, mean (SD)	31.1 (5.0)
Working up until pregnancy, yes, *n* (%)	244 (82.4)
Language ability, age‐corrected standard score, mean (SD)	105.8 (15.3)
Mental health at child's age of 12 months, mean (SD)	4.0 (3.3)

**TABLE 2:** The distribution of parent–child interactions per day at each wave of data collection (*n* = 296).

	5th percentile	25th percentile	50th percentile	75th percentile	95th percentile	Mean (SD)
6 months	138	222	325	434	559	331 (137)
12 months	151	253	343	481	643	368 (170)
18 months	156	323	468	691	1032	525 (289)
24 months	129	386	635	984	1531	702 (412)
30 months	119	471	666	987	1398	732 (385)
36 months	101	432	689	947	1400	708 (393)
42 months	141	471	656	940	1446	717 (382)
48 months	126	445	704	988	1486	741 (388)

*Note*: This is based on the distribution of the imputed data.

Models 1 and 2 estimates from the MSM are presented in Table S3, which were used in the modelling of the joint ATE. Table 3 presents the corresponding joint ATE and the 95% confidence intervals estimating the counterfactual outcome of the child's language development score, given a hypothetical scenario where the exposure trajectory was fixed to the *X*th percentile at each wave. For Model 1, the results indicate that if the entire population engaged in the bottom 5th percentile of parent–child interactions at each wave (see Table 2 for a description of the number of interactions observed at each percentile), the child's estimated counterfactual average language development at 36 months would be 100.42 (95%CI 100.11, 100.74). If the entire population of children increased their parent–child interactions to that observed at the 25th percentile at each wave their language outcome would increase to 101.83 (95%CI 101.51, 102.14). Language development continues to increase with higher exposure to parent–child interactions, reaching a standard score of 108.51 (95% CI 108.19, 108.82) at 36 months if the entire population had been exposed to the 95th percentile of parent–child interactions at each wave.

**TABLE 3:** The counterfactual language development standard score (CELF‐P2) that would occur if the whole population spoke *X* number of parent–child interactions at each time point after accounting for the time‐varying exposures and confounding (*n* = 296).

	36 months		48 months	
	Counterfactual CELF‐P2 standard score	95% CI	Counterfactual CELF‐P2 standard score	95% CI
5th percentile	100.42	100.11, 100.74	97.21	96.86, 97.56
25th percentile	101.83	101.51, 102.14	99.97	99.62, 100.32
50th percentile	102.80	102.49, 103.12	102.15	101.80, 102.50
75th percentile	104.72	104.41, 105.04	105.52	105.17, 105.87
95th percentile	108.51	108.19, 108.82	111.41	111.06, 111.76

The results for Model 2 demonstrate a similar pattern as seen in Model 1, with increases in a child's language development at 48 months when the entire population is exposed to increasing levels of parent–child interactions over time. If the entire population engaged in parent–child interactions observed at the 5th percentile at each wave, the child's estimated average language development at 48 months would be 97.21 (95%CI 96.86, 97.56), this would increase to 102.15 (95% 101.80, 102.50) at the 50th percentile and 111.41 (95% CI 111.06, 111.76) at the 95th percentile.

## 4 Discussion

This study used data from a prospective cohort study to answer a policy‐relevant question, concerning the causal effect of increasing the number of parent–child interactions on children's language development at 36 and 48 months. Findings provide evidence of a dose–response relationship, whereby incremental increases in exposure to parent–child interactions throughout the early years lead to increase in language development, but these effects were small. For example, the population's language development score at 36 months increased from 100.42 (5th percentile) to 102.80 (50th percentile) and to 108.51 (95th percentile) under the different hypothetical scenarios. The effects were slightly larger at 48 months with language development scores increasing from 97.21 (5th percentile) to 102.15 (50th percentile) and 111.41 (95th percentile). By modelling these hypothetical scenarios, it highlights the potential effect a real‐world intervention could have by increasing the number of parent–child interactions throughout the early years.

Identifying existing interventions that shift parent–child interactions can help us understand the potential benefit. One such intervention, trialled within local libraries in the United States for children aged 0–36 months (Beecher and Van Pay 2020), used LENA technology to provide language feedback to parents along with education on language‐promoting strategies. In their matched case–control study, the authors found an effect of changes in parent–child interactions; on average parent–child interactions in intervention participants increased by 150 over the 9‐week program, compared to a reduction of 45 interactions for controls. In the current study, this aligns with an estimated increase from the 5th percentile (138 interactions) to just below the 50th percentile (325 interactions) for children at 6 months old, equating to a potential 0.32SD increase in children's language development at 48 months if the increase in parent–child interactions was sustained.

These findings provide evidence for policymakers and early childhood professionals that investment in interventions that are cost‐effective at increasing the quantity of parent–child interactions may improve children's language development. It also reinforces the need for professionals who engage with families within the first year of life to be encouraging parents to engage in back‐and‐forth interactions with their baby, even before the child is talking themselves. Future research needs to understand specific intervention components, delivery methods and implementation considerations needed to support sustained increases in parent–child interactions across the early years, given the limited data on existing long‐term interventions. Given previous evidence, it is also possible that targeted interventions that promote parent–child interactions, and are tailored to the needs of the families, may demonstrate greater benefits for families known to be at risk of poor language development (Levickis et al. 2022; Zubrick et al. 2015). Future research should investigate the appropriate mix of universal and targeted support services to ensure all children receive equitable support with their early language.

The effects estimated through the hypothetical scenarios in the study were small and should be considered within the context of the outcome and exposure measures used. The language assessment, CELF‐P2, is designed to determine whether a language impairment is present among preschool‐aged children (Wiig et al. 2006). The LiLO study was focused on the language experiences of typically developing children and therefore excluded children with a diagnosed language difficulty at the outset and as diagnosis became apparent. The difficulties of existing language measures not being sensitive enough to detect intervention effects are a challenge within the field (Frizelle et al. 2023). Similarly in this study, this outcome measure may not have been sufficiently sensitive to capture subtle changes in language development in the general population. Furthermore, the exposure only measured one aspect of parent–child interactions (the number of conversational turns). Evidence indicates that the quality of interactions (i.e., complexity and diversity of vocabulary or grammar) may be more important than the quantity (Anderson et al. 2021). An intervention that aimed to promote both the quantity and quality of parent–child interactions across the early years may see greater effects on children's language development and should be a focus of future research. Future research could also undertake a cost‐benefit analysis to determine the potential return on investment at a population level, for an intervention that demonstrates small improvements in early language development.

This study is the first to use observational data, with objective measures of the home language environment, to estimate the causal effect of parent–child interactions across the early years on children's later language development. To our knowledge, the key confounding factors that may impact parent–child interactions and child's language development are included in analyses, but it remains possible that unmeasured confounding may be present, leading to potentially biased estimates. It was also out of scope within the current study to unpack the complex relationship between changes in the child's linguistic skills and increasing parent language input. Another limitation is that our sample was restricted to English‐speaking families, which may limit the generalisability of our findings for different language backgrounds or dual‐language learners.

## 5 Conclusion

Children's early language development is critical to their success in life, and identifying potential mechanisms that support language development offers opportunities to intervene. This study provides the first causal evidence, using observational data, to describe the impact of early parent–child interactions on children's later language development at 36 and 48 months. It highlights the importance of early intervention and long‐term investment in supporting parent–child interactions across the early years for beneficial impacts on later outcomes.

## Ethics Statement

Ethics approvals were granted by the University of Western Australia Human Research Ethics Committee (2022/ET000028).

## Consent

Written informed consent was provided by all participants in the study and by the parent or guardian for participants under 16 years old.

## Conflicts of Interest

The authors declare no conflicts of interest.

## Supporting information



Supporting Information

## Data Availability

The data that support the findings of this study are available from the corresponding author upon reasonable request.
