# Supplementary material for: The Causal Effect of Parent–Child Interactions on Child Language Development at 3 and 4 Years
Source: Int J Lang Commun Disord. 2025 May 5;60(3):e70045. doi: 10.1111/1460-6984.70045 (PMC12051723; doi:10.1111/1460-6984.70045)
Supplement: Supplementary file 1 — Supporting Information [file JLCD-60-0-s001.docx]

Supplementary Material

**Directed Acyclic Graph**

Time-varying confounders

*L*_1_

Time-varying confounders

*L*_2_

Time-varying confounders

*L*_8_

[…]

Baseline confounders

*C*

Parent-child interactions

*A*_1_

Parent-child interactions

*A*_2_

Parent-child interactions

*A*_8_

Language Development

*Y*

[…]

**Figure S1.** Directed Acyclic Graph (DAG) depicting the causal relationship between parent-child interactions (A) and the child’s language development (Y). The exposure, parent-child interactions, were measured at each time point (A_1_ … A_8_). Language development is the outcome measure (Y), at 36 and 48 months. Baseline confounders (C) include child gender, highest level of completed maternal education, child first born status, and primary caregivers language ability. Time-varying confounders (L_1_ … L_8_) include average number of home activities completed with the child, number of children’s books in the home, primary caregivers’ mental health, financial stability of the family, and amount of time the child was exposed to a screen.

**Summary of confounding variables**

Baseline confounders were collected by the researcher asking the primary caregiver either at the time of recruitment or at the first wave of data collection.

**Child gender** was reported as either male or female.

**Maternal education** was categorised into two groups (high educated or low educated) based on the highest level of completed education. High educated included mothers who had completed a university degree and low educated included mothers without any post-secondary school qualification. Eligibility criteria of the LiLO study meant any mothers who had completed a certificate or diploma were ineligible to participate in the study.

**Child first born status** was categorised as Yes or No, based on the number of other children under the age of 18 years living at home with the participant child at baseline. If the primary caregiver reported 0, they were classified as ‘Yes’. If any number of children were reported, they would be classified as ‘No’.

**Primary caregivers’ language ability** was not collected via self-report, but instead via direct assessment of the primary caregiver. The caregiver’s language ability was assessed using the NIH Toolbox Picture Vocabulary Test version 2.0. This is administered in a computer-adaptive format, whereby the following question depends on the answer to the previous question. The caregiver was presented with an audio recording of a word and four photographic images on an iPad. They were required to select the picture that most closely matches the word’s meaning. The test takes less than 5 minutes to administer. The NIH Toolbox automatically provides the scoring for the test based on the details provided for the participant’s age and gender. An age-corrected standard score (based on the normative US population) of 100 (SD = 15) indicates average vocabulary ability for an adult of the same age. This age-corrected standard score was used as the measure of parents’ language ability.

Time-varying confounders were collected via self-report at the biannual home visits. Where missingness was present in the data for some of the time-varying confounders, this was dealt with using multiple imputation (see Statistical Analysis section for details of the approach and Figure S2 and Table S1 for a summary of missingness).

**The average number of home activities completed with the child** was captured at every wave of data collection. This measure included seven items asking parents, “In the past week, on how many days have you or someone in your family done the following with the child?” parents could then respond (0) None; (1) 1 or 2 days; (2) 3-5 days; or (3) 6-7 days. The home activities included book reading, telling stories, drawing pictures, musical activities, indoor games, outdoor games, and everyday activities (i.e., cooking or caring for pets). The average score (range: 0 – 3) for home activities was used as the home activities variable, with higher scores representing greater levels of home activities completed with the child. The measure and scoring were taken from the Growing Up in Australia Study, Wave 1 Parent 1 Interview (4 years).

**The number of children’s books in the home** was collected as one indicator of a language-rich home environment. Parents were asked to self-report: “What is the total number of books or eBooks in the family home for children under five years?”. The response options included: (0) None; (1) 1-10; (2) 11-20; (3) 21-30; (4) 30+. This item was asked at each wave of data collection, beginning at the third wave (when children were 18 months old). The number of children’s books was measured by the ordinal variable (range 0 – 4).

**Primary caregiver mental health** was assessed using the Kessler K6 at each wave of data collection, beginning at the second wave when the children were 12 months old. The Kessler K6 is a self-report measure of psychological distress, including six questions about the individual’s emotional state. Caregivers were asked, “In the past four weeks about how often…” with questions such as: “…did you feel nervous?”, “… did you feel hopeless?” and “…did you feel that everything was an effort?”. The response options to these questions include (0) None of the time; (1) A little of the time; (2) Some of the time; (3) Most of the time; and (4) All of the time. The continuous summed score of each question (range 0 – 24) was then used to measure the primary caregiver’s self-reported mental health or psychological distress within this thesis.

**Family financial hardship** was collected once a year (waves 2, 4, 6, and 8), whereby the primary caregiver was asked to report on any instances of financial hardship in the previous 12 months. This scale asked, “Over the last 12 months, due to shortage of money, have any of the following happened?” with seven questions concerning instances like not being able to pay bills, rent or mortgage on time, whether family members had gone without meals, and whether you have been unable to heat or cool your home. Parents were asked to self-report, providing answers ‘Yes’, ‘No’ or ‘Don’t Know’. The continuous summed score (range 0 – 7) of the number of ‘Yes’ responses to each question was used for the variable of family financial hardship. The scale and scoring were taken from the Growing Up in Australia Study, Wave 1 Parent 1 Interview (Infant).

**Children’s screen time** was measured objectively through a combination of the LENA technology and human coding, which was undertaken as part of a sub-study. As well as providing automated measures of AWC, CVC, and CTC, LENA can estimate the amount of time the child was exposed to TV or Electronic Noise during their recording day. However, the LENA device could not determine if this TV/Electronic Noise measure was from a screen-based device or other electronic equipment. Therefore, each 5-min segment of LENA audio where the technology flagged ‘TV/Electronic Noise’ during a child’s recording day was exported and listened to by trained researchers to code the audio segments as either “Screen Media”, “Music”, “Noise” or “Sleeping”. The categorisation was based on audio cues such as the identification of everyday noises (e.g., microwave beeping, car radio), media content theme songs or character voices, contextual conversations around the electronic noise (e.g., the child asks for an iPad, or parent mentions the child is asleep) and reports within accompanying parent-completed activity diaries for the recording day (e.g., parent states the child is watching TV). When multiple researchers could not identify the source of the electronic noise, it was categorised as “Unknown”. Research staff working on the sub-study were trained by a master coder and required to achieve 90% accuracy compared to the master coder on the categorisation of screen media before they could begin coding independently. This approach to measuring screen time is unique and was developed specifically for this sub-study. The “Screen Media” measure captures the total time (in minutes) the child was exposed to a screen-based device while awake on their LENA recording day. Given the additional coding of the LENA audio, this sub-study required additional consent from participants. Screen media was able to be captured at each wave of data collection for a sample of 220 families who consented to participate in the sub-study (see Brushe et al, 2023; Brushe et al 2024 for further details).

**Assumptions of the Potential Outcomes Framework for Causal Inference**

To make causal inferences using observational data, several assumptions must be met that often cannot be tested statistically but need to be justified based on theory or existing evidence. A comprehensive discussion of the potential outcomes framework and the following assumptions can be found in “Causal Inference: What If” by Hernán and Robins (2020).

Exchangeability

One of the main benefits of randomised controlled trials (RCT) is the randomisation of participants into intervention and control groups, to ensure any predictors of the outcome are equally distributed between the two groups (i.e.,the two groups are exchangeable). To achieve this using observational data, a key assumption of causal inference is that all potential confounders, that influence both the exposure and outcome, have been sufficiently well-measured and controlled for within the analysis, in order to achieve exchangeability across the potential outcomes. Exchangeability means that the counterfactual outcome and the actual treatment are independent, or $Y^{a}\perp\perp A$, for all values of $a$. This is usually achieved through the research teams theoretical understanding of the causal relationship and depicted using directed acyclic graphs (DAG). See Figure S1 for the DAG used in the current study. While exchangeability assumes there is no unmeasured confounding within the study, this is difficult to test empirically, and so conclusions drawn from observational data need to be cognizant of this limitation.

Positivity

The positivity assumption refers to the idea that the probability of an individual being assigned to each exposure condition is greater than zero and for each covariate combination required to meet exchangeability. If some combination of the exposure or confounders were impossible (e.g., a child would never be exposed to the intervention if they lived in a specific location) within the real world, this is considered a structural positivity violation. Sometimes random positivity assumptions can occur when the combination is possible, but not captured within the sample. This can often be explored within the data for binary exposures or confounders. For continuous exposures, as is used within the current study, positivity is assumed as it is impossible to determine when potentially infinite combinations of exposure assignment are possible. In case when we have multiple exposures (t = >15) this assumption will be violated irrespective of the data in such situations one must be looking for more flexible models (see Rudolph et al., 2022).

Consistency

Consistency is the assumption that requires well-defined hypothetical interventions to avoid multiple different versions of the exposure with different causal effects. Specifically, consistency includes a sufficiently defined counterfactual outcome and the linkage of the counterfactual outcome to the observed outcomes. Whether a question is well-defined is determined by agreement among experts and based on currently available evidence.

**Missing Data**

Participants with at least 1 wave of data^1^

(n = 302)

Eligible sample, with missing data

(n = 296)

Imputed sample, with no missing data

(n = 296)

Participants with missing data on at least one exposure, confounder, or outcome across the study

(n = 166)

Participants excluded due to developing a diagnosed cause of language delay

(n = 6)

**Figure S2.** Flow chart reflecting exclusion and imputation for the final analysis sample.

^1^This number reflects the total number of participants who completed at least one wave of data collection between wave 1 (6 months) to wave 8 (48 months).

| **Table S1.** Summary of the amount of missing data in each variable from the eligible sample (n = 296) | |
| --- | --- |
|  | N(%) Missing |
| Exposures |  |
| Number of parent-child interactions, 6 months | 74 (25.0) |
| Number of parent-child interactions, 12 months | 57 (19.3) |
| Number of parent-child interactions, 18 months | 46 (15.5) |
| Number of parent-child interactions, 24 months | 37 (12.5) |
| Number of parent-child interactions, 30 months | 41 (13.9) |
| Number of parent-child interactions, 36 months | 59 (19.9) |
| Number of parent-child interactions, 42 months | 63 (21.3) |
| Number of parent-child interactions, 48 months | 81 (27.4) |
| Baseline Confounders |  |
| Mothers highest level of education | 0 (0.0) |
| Child gender | 0 (0.0) |
| Child gestational age | 0 (0.0) |
| Mothers age at childbirth | 0 (0.0) |
| Child first born status | 0 (0.0) |
| Mother in employment prior to pregnancy | 0 (0.0) |
| Primary caregivers’ language ability | 14 (4.7) |
| Time-varying Confounders |  |
| Number of minutes exposed to screens, 6 months | 121 (40.9) |
| Number of minutes exposed to screens, 12 months | 109 (36.8) |
| Number of minutes exposed to screens, 18 months | 100 (33.8) |
| Number of minutes exposed to screens, 24 months | 91 (30.7) |
| Number of minutes exposed to screens, 30 months | 85 (28.7) |
| Number of minutes exposed to screens, 36 months | 89 (30.1) |
| Home activities, 6 months | 72 (24.0) |
| Home activities, 12 months | 56 (18.9) |
| Home activities, 18 months | 43 (14.5) |
| Home activities, 24 months | 35 (11.8) |
| Home activities, 30 months | 29 (9.8) |
| Home activities, 36 months | 40 (13.5) |
| Home activities, 42 months | 54 (18.2) |
| Home activities, 48 months | 58 (19.6) |
| Primary caregivers’ mental health, 12 months | 56 (18.9) |
| Primary caregivers’ mental health, 18 months | 43 (14.5) |
| Primary caregivers’ mental health, 24 months | 35 (11.8) |
| Primary caregivers’ mental health, 30 months | 29 (9.8) |
| Primary caregivers’ mental health, 36 months | 40 (13.5) |
| Primary caregivers’ mental health, 42 months | 50 (16.9) |
| Primary caregivers’ mental health, 48 months | 58 (19.6) |
| Family’s financial stability, 12 months | 56 (18.9) |
| Family’s financial stability, 24 months | 35 (11.8) |
| Family’s financial stability, 36 months | 40 (13.5) |
| Family’s financial stability, 48 months | 58 (19.6) |
| Number of children’s books in the home, 18 months | 43 (14.5) |
| Number of children’s books in the home, 24 months | 35 (11.8) |
| Number of children’s books in the home, 30 months | 29 (9.8) |
| Number of children’s books in the home, 36 months | 40 (13.5) |
| Number of children’s books in the home, 42 months | 54 (18.2) |
| Number of children’s books in the home, 48 months | 58 (19.6) |
| Outcome |  |
| Child’s language development, 36 months | 72 (23.8) |
| Child’s language development, 48 months | 61 (20.6) |

*Note.* Screen time data was captured for all children who undertook a LENA recording day at each wave however, to undertake the manual coding of this data, additional consent from participant families was obtained retrospectively from 80.17% of participants to listen to segments of the LENA audio that were flagged as ‘electronic noise’. This explains the higher percentage of missing data on these variables, in comparison to other confounders.

|  | Eligible Sample |
| --- | --- |
| Child |  |
| Girls, n (%) | 157 (53.04) |
| Gestation, wk, mean (SD) | 39.25 (1.46) |
| Firstborn, n (%) | 148 (50.00) |
| Number of parent-child interactions at six months, mean (SD) | 331.28 (137.23) |
| Language development standard score at 48 months, mean (SD) | 104.32 (12.00) |
| Mother |  |
| Highest level of completed education, University, n (%) | 166 (56.08) |
| Age at childbirth, y, mean (SD) | 31.11 (4.96) |
| Working up until pregnancy, yes, n (%) | 244 (82.43) |
| Language ability, age-corrected standard score, mean (SD) | 106.08 (14.92) |
| Mental health at 12 months, mean (SD) | 3.86 (3.16) |

**Table S2.** Sociodemographic characteristics of the eligible sample, before imputation (n = 296).

**Fitting the Marginal Structural Mean Model**

*Step 1.* Fit a linear regression (e.g., linear as our exposure is measured continuously) between the exposure $(A)$ measured at time point, *t*, and the baseline confounders $(C)$. This will give the values of the predicted outcomes (i.e., the exposure within the context of this study). We then extracted the probabilities from the density curve using the observed mean of the exposure computed from the data and using the predicted mean outcome and the root mean square error from the regression model. For this we used the *normalden* function in Stata 17. These probabilities were then used as the numerator in equation (1).

*Step 2.* Fit a suitable regression between the exposure $(A)$ measured at time point, *t*, the baseline confounders $(C)$ and the time-varying confounders $\left( L \right)$, depicted as $\bar{L}$. Like step-1 the probabilities are extracted from the density curve and used in the denominator of equation (1).

*Step 3.* Use the numerator and denominator probabilities to create a stabilized inverse probability of treatment weight (IPTW) for the exposure at each individual time period.

*Step 4.* Repeat Steps-1-3 for all exposures. For the weight creation of exposures measured at $t+1$ we include the exposures measured at previous times as confounders in the regressions. To create the overall stabilized IPTW, we take the product of all the exposure weights measured from time point 1 until the final time point.

| (1) | $SW\left( t \right)= \prod_{k=0}^{t} \frac{f\left[ A\left( k \right) \right\vert\overline{A}\left( k-1 \right), C]}{f\left[ A\left( k \right) \right\vert\overline{A}\left( k-1 \right), \overline{L}(k)]}$ |
| --- | --- |

*Step 5.* Fit a regression using the outcome, the exposures measured from all time points and the baseline confounders. This regression is then weighted using the stabilised IPTWs created using equation (1).

**Fitting the Scenario setting of the joint ATE**

*Step 1.* Obtain the estimated beta coefficients ($\hat{\beta}$) of the outcome regression from the MSM described in the section above.

*Step 2*. The potential outcome ($Y^{PO}$) corresponding to the median value (for example, 325 interactions at the first time point, see Table 2) of the exposure measured at each time point was used as

$Y^{PO}=\hat{\beta_{0}}+\hat{\beta_{1}}*325+\hat{\beta_{2}}*343+\hat{\beta_{3}}*467.5 [\ldots]+\hat{\beta_{8}}*704+\hat{\beta_{9}}C_{1}+\hat{\beta_{10}}C_{2} +\hat{\beta_{11}}C_{3}+\hat{\beta_{12}}C_{4}$

Similarly other potential outcomes were modelled by replacing the median value of the exposure at each time point with the value observed at the 5^th^ percentile, 25^th^ percentile, 75^th^ percentile, and the 95^th^ percentile in the observed distribution (see Table 2). The distribution of the baseline confounders ($C_{1-4})$were assumed to take the natural value that was observed in the original data.

*Step 3.* Using the estimated potential outcome mean and the standard deviations, confidence intervals were computed using a normality assumption.

| **Table S3.** Marginal structural model results after accounting for measured confounding and time-varying exposures using stabilised IPTW (n = 296). | | | | | | | | | |
| --- | --- | --- | --- | --- | --- | --- | --- | --- | --- |
|  | Language Development at 36 months  (Model 1) | | | | | Language Development at 48 months  (Model 2) | | | |
|  | β | | 95% CI | | *p-value* | β | 95% CI | | *p-value* |
| Intercept | 83.41 | 72.73, | | 94.08 | 0.00 | 79.72 | 69.27, | 90.16 | 0.00 |
| Parent-child interactions, 6m | -0.01 | -0.02, | | -0.00 | 0.04 | -0.00 | -0.01, | 0.01 | 0.98 |
| Parent-child interactions, 12m | -0.00 | -0.01, | | 0.01 | 0.38 | -0.01 | -0.02, | -0.00 | 0.01 |
| Parent-child interactions, 18m | 0.02 | 0.01, | | 0.02 | 0.00 | 0.02 | 0.01, | 0.02 | 0.00 |
| Parent-child interactions, 24m | 0.00 | -0.00 | | 0.01 | 0.61 | 0.00 | -0.00, | 0.01 | 0.16 |
| Parent-child interactions, 30m | -0.00 | -0.00 | | 0.00 | 0.66 | -0.00 | -0.00, | 0.00 | 0.89 |
| Parent-child interactions, 36m | 0.00 | -0.01 | | 0.01 | 0.74 | 0.00 | -0.00, | 0.01 | 0.39 |
| Parent-child interactions, 42m |  |  | |  |  | 0.00 | -0.00, | 0.01 | 0.17 |
| Parent-child interactions, 48m |  |  | |  |  | -0.00 | -0.01, | 0.00 | 0.11 |

*Note.* A one-unit increase in parent-child interactions reflects one additional conversation between parent and child.

**A note of caution in interpreting the MSM model**

The MSM estimates at each time point can be interpreted as the mean change in the child’s language development score that would be observed at 36 or 48 months if every child had a one-unit increase in parent-child interactions at that specific time point, holding all prior and future exposures and baseline confounders constant, versus the level of parent-child interaction they actually received at that time point. These estimates are known as the controlled direct effects, and form only part of the total effect. These are not meaningful estimates themselves as they cannot be interpreted as time-specific total effects but are a necessary step in understanding the joint ATE. These causal estimands are important in that they create a pseudo population which has taken into account the time-varying exposures and confounding. The β coefficients are then used in step two of the modelling approach to estimate the joint ATE of the different hypothetical scenarios.
